# The phenanthrene derivative PJ34 exclusively eradicates human pancreatic cancer cells in xenografts

**DOI:** 10.18632/oncotarget.27268

**Published:** 2019-10-22

**Authors:** Leonid Visochek, Dikla Atias, Itay Spektor, Asher Castiel, Talia Golan, Malka Cohen-Armon

**Affiliations:** ^1^ Sackler Faculty of Medicine, Tel-Aviv University, Tel-Aviv 69978, Israel; ^2^ Sagol School of Neuroscience, Tel-Aviv University, Tel-Aviv 69978, Israel; ^3^ Oncology Institute, Sheba Medical Center, Ramat Gan 53621, Israel

**Keywords:** PJ34, pancreas cancer, stroma, PANC1 cancer xenografts

## Abstract

Recent reports demonstrate an exclusive eradication of a variety of human cancer cells by the modified phenanthridine PJ34. Their eradication during mitosis is attributed to PJ34 preventing NuMA clustering in the mitotic spindle poles of human malignant cells, which is crucial for their normal mitosis. Here, the effect of PJ34 is tested in cell cultures and xenografts of human pancreas ductal adenocarcinoma. Evidence is presented for a substantial reduction (80–90%) of PANC1 cancer cells in xenografts, measured 30 days after the treatment with PJ34 has been terminated. Benign cells infiltrated into the PANC1 tumors (stroma) were not affected. Growth, weight gain and behavior of the treated nude mice were not impaired during, and 30 days after the treatment with PJ34. The efficient eradication of malignant cells in human pancreas cancer xenografts presents a new model of pancreas cancer treatment.

## INTRODUCTION

Despite a substantial advance in cancer treatment, pancreatic ductal adenocarcinoma (PDAC) have a limited response to current treatments, and a low 5-years survival rate of about 6% [1–3]. Thus, there is an urgent need to explore new mechanisms for treating this lethal malignancy.

Recent reports have discovered the capability of phenanthrenes to kill human cancer cells that are resistant to currently prescribed apoptosis-inducing agents [4, 5]. Furthermore, we identified phenanthrenes acting as PARP1 inhibitors that efficiently eradicate a variety of human cancer cells without impairing benign cells [6–9]. Notably, their exclusive cytotoxic activity in human cancer cells was independent of, and un-related to PARP1 inhibition [7–11]. The phenanthrenes PJ34, TiqA and phenanthridinon (Phen) act as PARP1 inhibitors due to their binding potency to the nicotine-amide binding site in the catalytic domain of PARP1 [12, 13]. However, their PARP1 inhibition per-se does not impair nor eradicate human malignant cells, including pancreas cancer cells, PANC1 [7–9]. In contrast, at higher concentrations than those causing PARP1 inhibition, PJ34, Tiq-A and Phen eradicate a variety of human cancer cells by ‘mitotic catastrophe cell death’. This cell-death follows mitosis arrest caused by preventing the post translational modification of NuMA (Nuclear mitotic apparatus protein-1) that enables its binding to proteins [8].

In the tested human cancer cells, NuMA binding to proteins enables its clustering in the spindle poles, which is crucial for stabilizing the spindle, a pre-requisite for chromosomes alignment in the spindle mid-zone and normal anaphase. Notably, NuMA silencing or down regulation of NuMA prevents mitosis in all cell types [14–17].

In human malignant cells, specific post translational modifications of NuMA enable and promote NuMA binding to proteins [18–21]. These post translational modifications are most efficiently prevented by PJ34 [8, 20, 21]. In accordance, in PJ34 treated cancer cells, NuMA is arbitrarily dispersed in the spindle, instead of being clustered in the spindle poles [8]. The consequences are un-stabilized spindle pole with dispersed chromosomes, instead of segregated chromosomes aligned in the spindle mid-zone [8, 17, 22, 23]. This abnormality jeopardizes normal ploidy of the ‘daughter’ cells and evokes mitosis arrest [17, 23]. Cells with these abnormal spindles are eradicated by a rapid death mechanism, ‘mitotic catastrophe’ cell-death [9, 23].

Here, the efficacy of PJ34 to eradicate human pancreas cancer cells is tested in cell cultures and in xenografts. PANC1 cells are most frequently identified in human pancreas tumors [1–3]. Pancreas xenografts were developed in nude mice. These tumors also contained normal infiltrating cells (stroma) of mouse origin [24–27], i. e. fibroblasts, myofibroblasts, macrophages and lymphocytes infiltrated into the tumors [25, 26].

Mitosis arrest and cell death were measured in PANC1 cells incubated with PJ34. In xenografts, eradication of human PANC1 cells deduced from a massive reduction of human proteins in the tumors, was measured 30 days after the treatment with PJ34 has been terminated. An increased necrosis measured in the PANC1 tumors of mice treated with PJ34 supports cell-death caused by PJ34 in the xenografts. Normal cells infiltrated into the tumors were not impaired by PJ34. A similar cytotoxic activity of PJ34 was observed in patients-derived PDAC cells and xenografts. These results indicate the potency of PJ34 to eradicate human pancreas cancers.

## RESULTS

### Treatment with PJ34 causes mitosis arrest and cell death in human pancreas cancer cells PANC1

**Figure 1:**
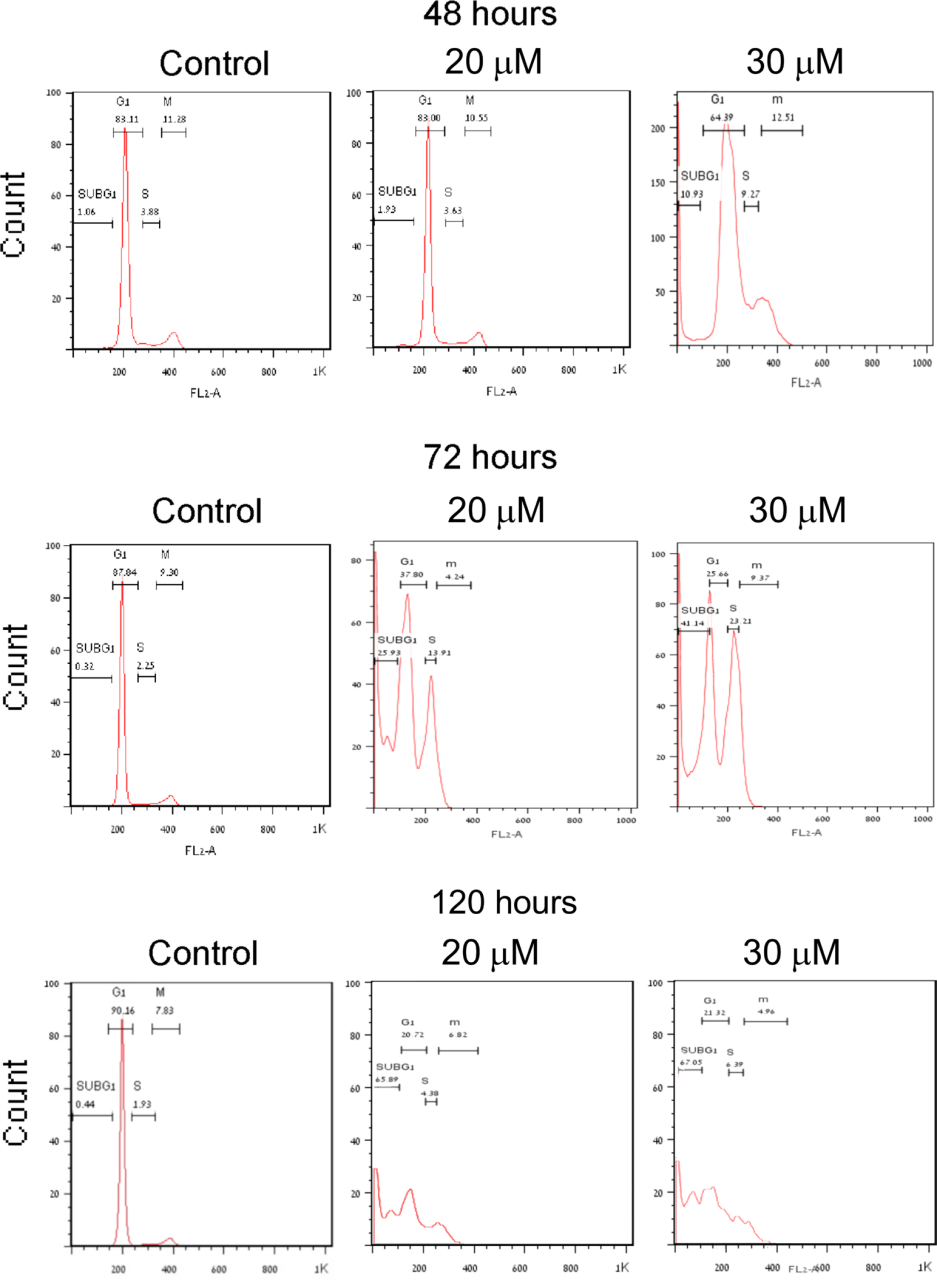
Mitosis arrest preceding cell death in human PANC1 cells treated with PJ34. PANC1 cells, 24 hours after seeding, were incubated with PJ34 at the indicated concentrations. Changes in PANC1 cells ploidy were monitored by flow cytometry (Methods). The effect of PJ34 on the percentage of cells at each ploidy state (indicating the kinetics of S-phase entry and G2/M transition and sub-G1-dead cells) is quantitated by this method. Piled-up cells in double ploidy state after 48–72 hours incubation with PJ34 indicates cell-cycle arrest before or in mitosis. A massive cell death is measured after 120 hours incubation with PJ34.

Measuring changes in the ploidy of PJ34 treated PANC1 cells with stained DNA by flow cytometry reveals piled up PANC1 cells with double DNA content, unable to proceed to mitosis (Figure 1). A similar cytotoxic effect of PJ34 is measured in other human malignant cell types [7]. Recently, the molecular mechanism causing mitosis failure and arrest in human cancer cells incubated with PJ34 has been disclosed [8]. The cell cycle profile of PANC1 cells incubated with PJ34 reflects their mitosis arrest and cell death (Figure 1). PJ34 (20 μM or 30 μM) was applied 24 hour after seeding, and PANC1 cell eradication and the kinetics of S-phase entry and G2/M transition were measured by flow cytometry after 48, 72 and 120 hours incubation (Methods). After 48 hours incubation with PJ34, failure to proceed into mitosis preceded cell death measured after 72 hours incubation. These cells were eradicated after 120 hour incubation with PJ34 (Figure 1).

### PJ34 efficacy tested in human PANC1 xenografts

**Figure 2:**
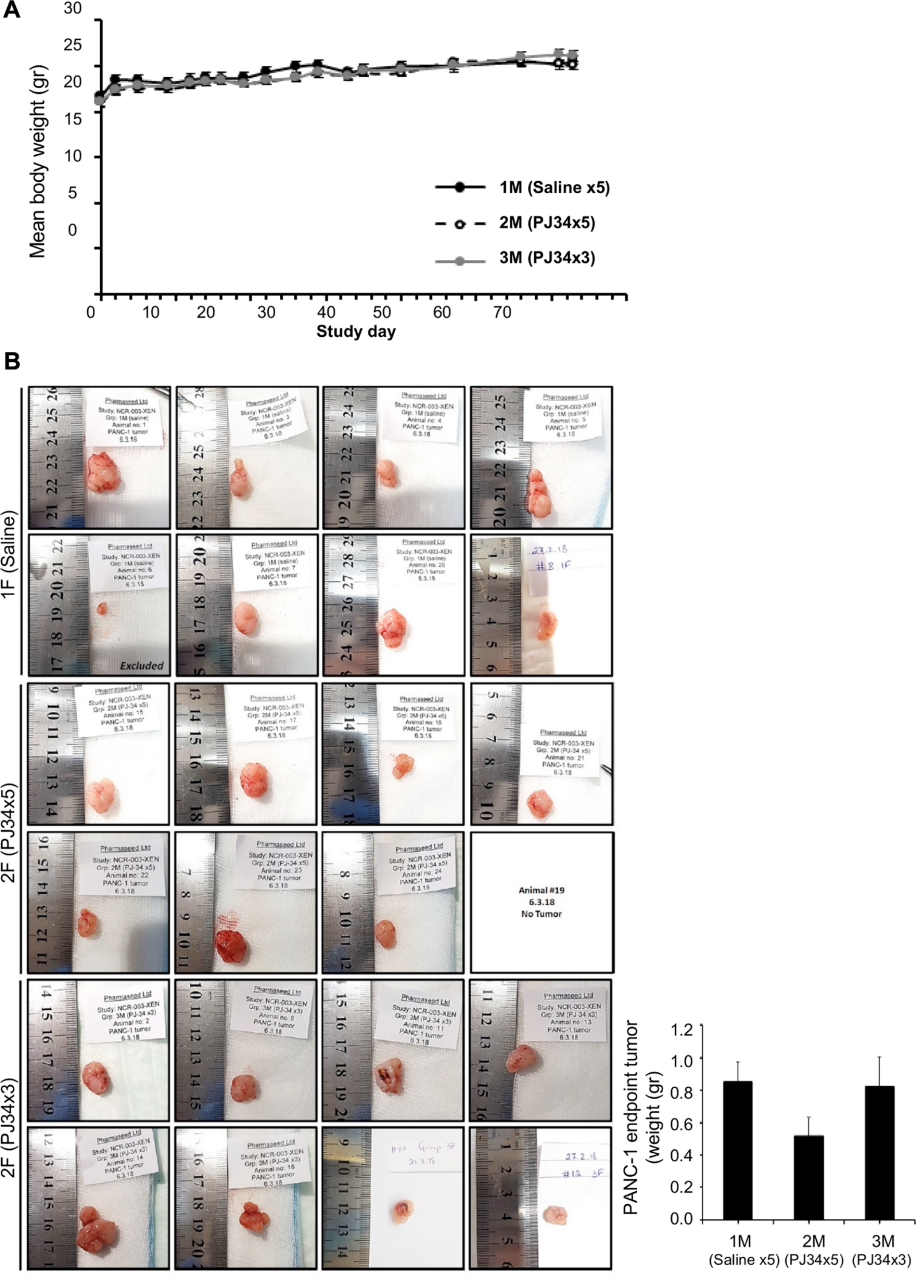
PJ34 treatment during the development of PANC1 xenografts. (**A**) Treatment with PJ34 did not impair the weight gain of nude mice developing PANC1 tumors. PJ34 was injected IV (60 mg/Kg dissolved in 100 μl saline, approximately 1 mg PJ34 per mouse). Control nude mice were injected daily with saline. A similar weight gain was recorded in untreated mice (control) and in mice treated 3 weeks with PJ34, either daily (5 days a week), or every second day (3 times a week). (**B**) PANC1 tumors were excised at the end of the study, 30 days after the treatment with PJ34 has been terminated. Left: Pictures of the tumors in the numbered mice. In mouse # 6 of the control group, tumor did not develop. In mouse #19 of the PJ34 daily treated group, tumor disappeared on day 56 of the study. Right: Calculated average weight of the tumors in the control group and in the two groups of mice treated with PJ34.

PJ34 evoked cell death in PANC1 cells has been further examined in PANC1 xenografts. The course of the experiment was based on previous experiments with PANC1 xenografts [28]. A total of 24 nude mice were randomly divided into 3 groups of 8 mice each. PANC1 cells in cell cultures were subcutaneously injected (5 × 10^6^ cells per mouse; Methods). Treatment with PJ34 started once PANC1 tumors reached a mean volume of approximately 100 mm^3^ (about 15 days after mice were injected with PANC1 cells). Nude mice of one group were injected intravenous (IV) with saline, daily 5 days a week for 3 weeks (control group). In the second group, nude mice were injected IV with PJ34 dissolved in saline, daily 5 days a week for 3 weeks. In the third group, nude mice were injected IV with PJ34 in saline, 3 times a week, every second day, for 3 weeks. Each injected dose contained 60 mg/Kg PJ34 in 100 μl saline (about 1 mg PJ34 per mouse). The volume of the developing tumors and the mice’ weight were monitored throughout the experiment (Figure 2A). In previously described experiments with PANC1 xenografts, PANC1 tumors were developed during 60 days [28]. In our experiments, on day 63 of the study, and 30 days after terminating the treatment with PJ34, mice were euthanized. Their excised tumors were measured and prepared for immuno-histochemichemistry. During the 30 days after treatment with PJ34 has been terminated, mice were not treated with any additional treatment.

No abnormalities, toxic signs, or animal death were observed during the study. On the contrary, all mice gained weight during the study, with no difference between control and the two PJ34 treated groups, evidence for wellbeing of the treated mice (Figure 2A). In support, no toxicity has been found in an additional study with immunocompetent BALB/C mice, IV injected with even higher doses of PJ34. These mice were examined for 14 days after treatment. No signs of toxicity were detected, and PJ34 did not impair their weight gain (Supplementary Figure 1).

At the end of the study with the immunodeficient (nude) mice, only the group treated daily with PJ34 (5 times a week) showed a significant reduction of about 40% in tumor size (volume and weight, Figure 2B). In addition, in one treated mouse in this group, the tumor started to shrink on day 35 and disapeared on day 56 (Figure 2B, mouse #19). Furthermore, immunohistochemistry conducted in the excised tumors of all the tested mice revealed a massive reduction in human proteins and increasing necrosis in the tumors of mice treated with PJ34.

**Figure 3:**
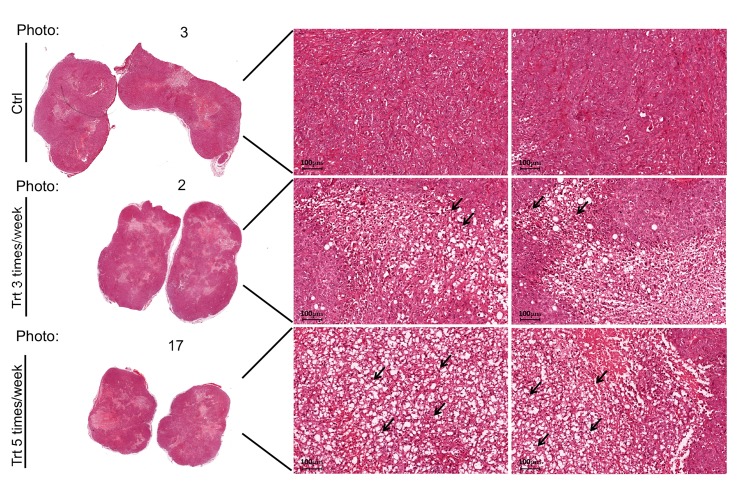
PJ34 induces necrosis in PANC1 tumors of the treated mice. Representative necrotic areas in Haemotoxylin and Eosin stained slices of PANC1 tumors, 30 days after the treatment with PJ34 (60 mg/Kg) has been terminated. Necrosis developed in the tumors of PJ34 treated mice, either the daily treated or treated 3 days a week. Representative results in two slices prepared from each tumor developed in the control and PJ34 treated mice are displayed. Arrows point at necrotic areas in the slides. Stained slices of each tumor were examined under light microscope. Magnification: 20×, Scale bar: 100 μm.

**Figure 4:**
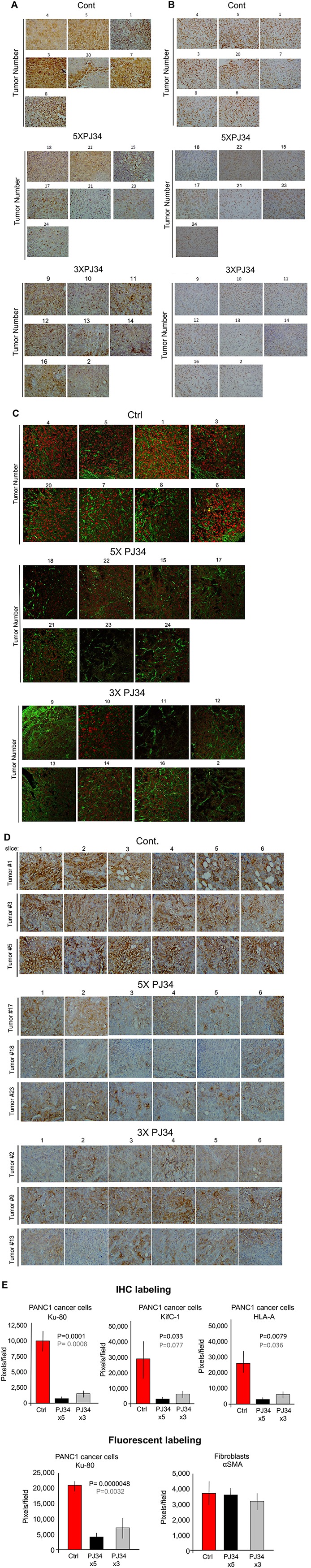
A massive reduction of human proteins in PANC1 xenografts in response to treatment with PJ34. Three arbitrarily selected human proteins were specifically immunolabeled in PANC1 tumors developed in nude mice of 3 tested groups, control and treated with PJ34, 5 times, or 3 times a week (PJ34, 60 mg/Kg in 100 μl saline). Proteins were specifically immuno-labeled in the excised tumors, 30 days after the 3 weeks treatment with PJ34 has been terminated. Immuno-labeling with specific antibodies directed against human HSET/kifC1 (**A**), the c-terminal of human Ku-80 protein (**B** and **C**) and Human Leucocytes Antigen (HLA) (**D**) is displayed. In (**C**) fluorescent immuno-labeling of human Ku-80 (red) and the Smooth Muscle Actin (aSMA) (green; expressed in rodents and human fibroblasts) are displayed. (**E**) A quantitative analysis of the immuno-labeling indicates a reduction of 80–90% with a high statistical significance in the indicated human proteins (**A**–**D**) in tumors of PJ34 treated mice versus control mice. Immuno-labeling of aSMA was hardly affected by the treatment with PJ34. Control: red columns, PJ34 treated: 5 times a week-black columns, 3 times a week-grey columns.

The excised tumors were sliced and prepared for histochemical analysis (Methods). Hematoxylin and Eosin labeling of the slices revealed higher necrosis in the tumors developed in PJ34 treated nude mice (Figure 3). In addition, the amounts of arbitrarily selected three human proteins were measured 30 days after the treatment with PJ34 has been terminated (Figure 4). Immunolabeling of these human proteins in all the tumors revealed that both treatment regiments with PJ34 (daily and 3 times a week) caused 80–90% reduction in their amount in the tumors. The daily treatment provided better results with higher statistical significance (Figure 4E). PANC1 cells were the only human cells in the PANC1 xenografts. Thus, the similar and substatial reduction in the amount of three arbitrarily selected human proteins, 30 days after the treatment with PJ34 has been terminated is attributed to the eradication of PANC1 human cells in the tumors of nude mice treated with PJ34 (Figure 4). This is supported by the enhanced necrosis in tumors of mice treated with PJ34, and by the massive eradication of PANC1 cells incubated with PJ34 (Figures 1, 3 and [7, 8]). The relatively short turnover of proteins in the cell (hours), as well as the MRT (mean residence time, 41 min) of PJ34 in mice contradict a possible effect of PJ34 on the expression of the three arbitrarily selected human proteins, 30 days after the treatment with PJ34 has been terminated. In addition, there was no reduction in the amount of an abundant protein in fibroblasts infiltrated into tumors of mice treated with PJ34, also contradicting an assumption of some general effect of PJ34 on protein expression 30 days after the treatment with PJ34 has been terminated.

The specific immunolabeling of each of the three proteins was measured in 3-4 different slices of each tumor, immunolabeled with specific antibody (Methods). Antibodies were directed against the human kinesin HSET/KifC1, or the c-terminal residue of the human nuclear protein Ku-80, or the Human Leucocytes Antigen (HLA), frequently found on the membrane of human cancer cells (Figure 4A–4D). Measurements of PJ34 induced changes in the immune-labeling of the three proteins provided results with a higher statistical significance. Normal cells infiltrated into the pancreas tumors (stroma) were immuno-labeled against smooth muscle actin αSMA that is mainly expressed in normal fibroblasts of both human and rodents [24, 25] (Figure 4C). The benign cells infiltrating into the human PANC1 xenografts were probably of mouse origin [26, 27]. In each slide, 6–8 different ‘fields’ were analysed (Methods; Figure 4E). Abnormal human tissues that may comprise metastasis were not found in the liver or guts, neither in treated nor in the untreated control mice. This fact is in accordance with other signs indicating the wellbeing of all the mice in this study (Figure 2A).

### PJ34 efficacy tested in patients-derived pancreas cancer cells and xenografts

**Figure 5:**
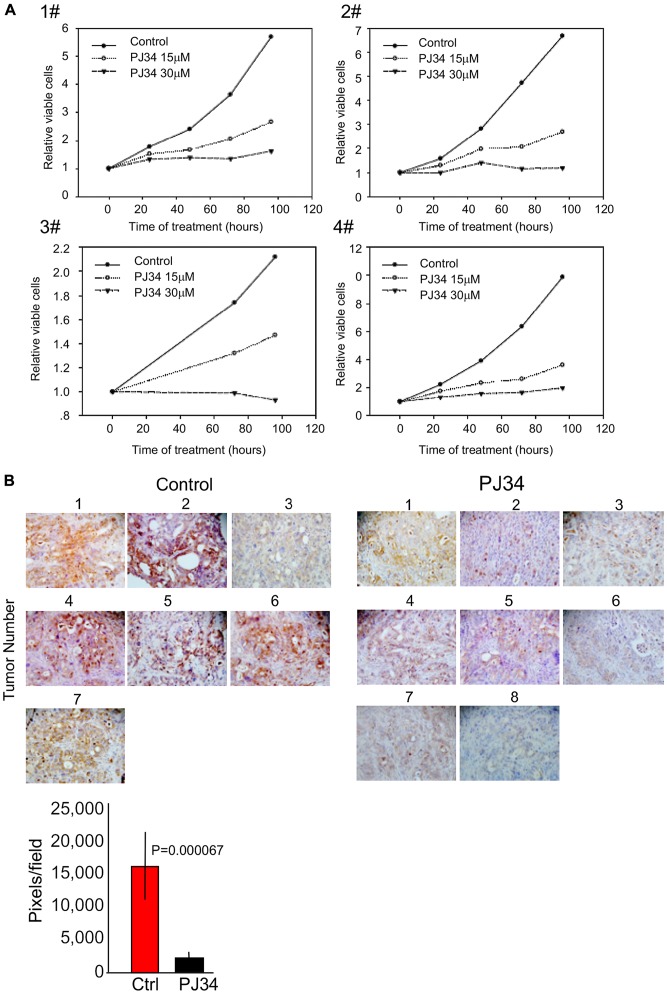
PJ34 cytotoxicity in patients-derived pancreas cancer cells. (**A**) PJ34 cytotoxicity in cell culture prepared from patients-derived xenografts. Cell cultures derived from four different types of pancreas cancer xenografts were incubated with PJ34 15 μM and 30 μM, applied 24 hours after seeding. Cell survival was quantified after 24, 48, 72 and 96 hours incubation with PJ34. The effect of PJ34 on cell viability was measured by the Sulforhodamine B (SRB) cytotoxicity assay (Methods). Each sample was tested in triplicate, and the displayed results are representative of three independent experiments. (**B**) The efficacy of PJ34 tested in xenografts derived from ascites/pleural effusion of pancreas cancer patient #1 (Methods). Mice (8) were injected I. P. with PJ34 (60 mg/Kg in saline, 5 days a week for 3 weeks). The human kinesin HSET/KifC1 specifically immuno-labeled (brown) in the excised tumors is displayed. A quantitative analysis of its immuno-labeling indicates about 90% (*p* = 0.000067) reduction in the quantity of HSET/kifC1 in tumors of mice treated with PJ34 in comparison to their quantity in tumors of untreated mice.

In view of evidence indicating eradication of PANC1 cancer cells by PJ34 (Figures 1 and 4), the effect of PJ34 was further tested in other types of patients-derived pancreas cancer cells. Ascites/pleural effusion derived cells were prepared from 4 different deceased pancreas cancer patients in the Sheba Medical Center [1] (Methods). These cells did not include PANC1 cells. Samples of the effusion fluid were injected subcutaneously in nude mice, and cell cultures were prepared from the developed tumors. PJ34 (15 and 30 μM) added to these cell cultures, 24 hours after seeding has been tested for its effect on cell viability during 24, 48, 72 and 96 hours incubation. The effect of PJ34 was measured by the Sulforhodamine B (SRB) cytotoxicity assay (Methods). PJ34 dose-dependently reduced the cell counts in the four different patients-derived cell cultures (Figure 5A). Next, PJ34 has been tested in xenografts developed after injecting nude mice (8) with Acite/pleural effusion of pancreas cancer patient #1. After pancreas tumors reached a volume of about 100 mm^3^. treatment with PJ34 started. Mice were injected daily with PJ34 (I. P.) 5 days a week for 3 weeks (60 mg/Kg PJ34, about 1 mg PJ34 in 100 μl saline per mouse). Untreated control nude mice were similarly injected IP with saline. The study ended 5 days after terminating the treatment with PJ34. Both control and PJ34 treated mice were euthanized and their excised tumors were measured and prepared for immuno-histochemichemistry (Methods). Human kinesin HSET/kifC1, HLA and human Ku-80 were specifically immunolabeled in all the tumors. About 90% reduction in the amount of HSET/kifC1 (*p* = 0.00067) and in Ku-80 (*p* = 0.00002) were measured in tumors developed in mice treated with PJ34 (Figure 5B). It was attributed to the eradication of the patients-derived pancreas cancer cells in the xenografts. Notably, treatment with PJ34 caused a similar reduction in HSET/KifC1 and Ku-80 labeling in PANC1 xenografts and in xenografts of pancreas patient#1 (Figures 4E and 5B).

## DISCUSSION

This study indicates the potency of PJ34 to cause a substantial eradication of pancreas cancer cells in xenografts. In addition to the measured moderate change in PANC1 tumors’ size, in one mouse (mouse # 19) the tumor started to shrink after 3 weeks of daily treatments with PJ34, and disappeared on day 56 of the study (Figure 2B). Furthermore, 30 days after the treatment with PJ34 has been terminated, a 80–90% reduction in human proteins in the tumors has been measured. Their small quantities is attributed to eradication of the human PANC1 cancer cells, the only human cells in the xenografts. Immuno-histochemistry performed in slices of all PANC1 tumors revealed the massive reduction in immunolabeled human proteins in the tumors developed in mice treated with PJ34, without affecting an abundant protein in fibroblasts infiltrated into the tumors (Figure 4). Thus, eradication of human PANC1 cells in the xenografts is deduced from the reduction in the measured (with a high statistical significance) immuno-labeling of three arbitrarily selected human proteins in PANC1 tumors developed in mice treated with PJ34, compared to their immunolabeling in tumors of untreated mice (Methods) (Figure 4E). A similar reduction in immuno-labeled human proteins was measured in a patients-derived pancreas cancer xenografts [1] (Figure 5B). The enhanced necrosis in PANC1 tumors developed in mice treated with PJ34 supports cell eradication in these tumors (Figure 3). Eradication of PANC1 cells in the tumors is also in line with PJ34-evoked cell death of PANC1 cells (Figure 1 and [7, 8]). An abundantly expressed protein in fibroblasts infiltrated in the PANC1 tumors was not affected in mice treated with PJ34 (Figures 4C and 4E). This is in accordance with previous reports [6–8]. Mesenchymal, endothelial and epithelial cells are not affected by the cytotoxic activity of PJ34 in cancer cells [7]. A molecular mechanism causing this exclusive cytotoxic activity of PJ34 in various human cancer cells including PANC1 has been recently identified [8].

A substantial volume of pancreas tumors is occupied by stroma [26, 27]. Thus, the exclusive eradication of PANC1 cells in the xenografts could be screened by un-affected cells in the stroma, causing a descrepancy between the modest reduction in the volume of PANC1 tumors developed in PJ34 treated mice versus the substatial reduction of PANC1 cells in these tumors (Figure 2B vs. Figure 4E).

The low immuno-labeling of the proteins in tumors of mice treated with PJ34 (Figure 4E) is not attributed to their impaired expression; The MRT (41 min) of PJ34, and the average short turnover of proteins in the cell (hours), contradict a possible effect of PJ34 on the expression of proteins in PANC1 cancer cells, measured 30 days after the treatment with PJ34 has been terminated. Furthermore, the substantial necrosis observed in tumors of mice treated with PJ34 supports cell death evoked by the treatment (Figure 3), in accordance with the eradication of PANC1 cells incubated with PJ34 (Figure 1 and [7]).

The effect of a daily treatment with PJ34 provided results with higher statistical significance than those obtained by the 3 times a week treatment with PJ34. However, both treatments caused a similar massive reduction of human proteins in the tumors, 30 days after the treatment with PJ34 has been terminated.

PJ34 concentration in the blood plasma raises up to 30 μM within 5 min after I. V. injection, and instantly starts decreasing towards zero. PJ34 is distributed within an hour in the mouse tissues (Vd, volume of distribution=3722 ml/kg, T1/2=155 min, Cl, clearance=179 ml/min/kg) (Supplementary Figure 2). Possible difference in the clearance, T1/2 and Vd in different mice, as well as the proximity of the developing tumor to blood vessels could cause differences in the effect of PJ34 in different mice, as the disappearance of PANC1 tumor after the treatment has been terminated only in mouse #19 out of a group of 8 mice, daily treated with PJ34. (Figure 2B). Since PJ34 is not cytotoxic in benign cells (Figure 4E and [6–8]), the disappearance of the tumor in mouse #19 also raises questions about a possible effect of PANC1 cells eradication on the presence of benign cells in the stroma. It is possible that benign cells infiltrated into the tumor in the presence of PANC1 cells, leave the tumor after PANC1 eradication. Pancreatic ductal adenocarcinoma tumors are characterized by a dense desmoplastic stroma and an inflammatory reaction [26]. In some research projects, targeting stromal cells seems to be an important therapeutic goal [27]. Different studies suggest that stromal desmoplasia promotes tumor progression, and is an obstacle to cytotoxic therapies [27]. It has been also suggested that benign cells of the stroma act differently in pancreas tumors depending on the tumor progression stage [27]. A better understanding of the interactions between benign cells in the stroma and PANC1 cancer cells, and their mutual effects on each other’s survival may better predict the consequences of an exclusive eradication of malignant PANC1 cells, and its effect on the presence of benign cells in the tumor. Thus, in addition to efforts aiming at understanding the role of stroma in malignancy propagation, the dependence of the benign cells on the presence of PANC1 cells in the tumor should be clarified when treatment with PJ34 is considered.

According to the present results, PJ34 does not seem to harm the wellbeing of the treated mice, or their weight gain (Figure 2A). In support, MTD (maximal tolerance dose) experiment performed with immunocompetent BALB/C mice IV injected with higher doses of PJ34 did not exert morbidity or mortality, nor indicated any abnormal clinical signs in the mice or their weight gain (Supplementary Figure 1). An additional advantage of the treatment with PJ34 is its potency to inhibit matrix metalloproteinases, which may decrease chances to develop metastases [29].

In conclusion, according to these results, a small molecule that prevents the clustering of NuMA in the mitotic spindle poles of human cancer cells, efficiently eradicates PDAC cells. PJ34, which is permeable in the cell membrane, accessed and eradicated human PDAC cells in xenografts without impairing normal proliferating cells infiltrated into the tumors. The exclusive cytotoxicity of PJ34 in human cancer cells offers a new model of pancreas cancer therapy which does not impair normal tissues.

## METHODS

Flow cytometry was used to monitor changes in the cell cycle of PANC1 malignant cells. At the indicated time points, PANC1 cells were collected, permeabilized (75% Ethanol in DDW), and their DNA was stained with Propidium Iodide. Cell death and the kinetics of S-phase entry and G2/M transition were monitored by measuring the changing percentage of cells at each ploidy level. Measurements were performed with Beckton Dickenson machine and the FlowJo software (https://www.flowjo.com/, three Star, Ashland, OR). Changes in the cell cycle were monitored during 120 hours incubation with PJ34 at various concentrations. This method has been used before [7].

### Measuring viability of patients-derived pancreas cancer cells

PDAC cells were prepared from patients-derived xenografts. They are referred as Sheba Pancreatic Cancer (SPC) [1]. These cells were cultured in RPMI-1640 media supplemented with 10% FBS, 2 mM L-glutamine, 1% penicillin streptomycin and 1% sodium pyruvate (Biological industries, Israel), and seeded at a density of 5 × 10^3^ cells/well in 96 well plates. Cells were incubated for 24–96 hours with 15 and 30 μM PJ34, applied 24 hours after seeding. Cell viability was assessed by the sulforhodamine B (SRB) assay, as previously described [30]. In brief, sulforhodamine B (SRB) assay is based on the measurement of the content of labeled cellular protein. At the indicated time, sampled cells were fixed with 10% trichloroacetic acid and stained with 0.057% SRB. The excess dye was removed by washing repeatedly with 1% (vol/vol) acetic acid. The protein-bound dye indicates proteins in viable cells adherent to the plats. It was dissolved in 10 mM Tris base solution for OD determination at 510 nm.

### PJ34 treatment of PANC1 xenografts

The *in-vivo* studies were approved by the National Animal Care and Use Committee. The experiments were performed by Pharmaseed pre-clinical, GLP approved CRO. Nude female mice were purchased from Envigo and maintained in specific pathogen-free (SPF) and temperature-controlled room at 20° C with a 12 h- dark-light cycle. The mice had free access to water and commercial chow. PANC1 cells (5 × 10^6^ cells) were injected in the lower flank of 6-weeks-old female nude mice. When tumors reached the size of ~100 mm^3^, mice were randomized into two treatment groups (*n* = 7–9): PJ34, 60 mg/kg in 100 μl saline was administered intra-venous (IV), either daily five days a week, or every second day, for 3 weeks. Vehicle control mice were IV injected daily with saline only, 5 days a week for 3 weeks. All mice were maintained without treatment for an additional month after the treatment with PJ34 has been terminated. The mice weight was monitored during the study and the developing tumors were monitored during the study by a caliper according to the formula (length × width^2^)/2. PJ34 60 mg/Kg is the maximal dose we tested. This I. V. dose increased the concentration of PJ34 in the blood plasma within 5 min to 30 μM. At this concentration PJ34 does not impair a variety of benign cells, but efficiently eradicates a variety of resistant cancer cells [6–9]. The PJ34 concentration in the blood plasma of the treated mice instantly dropped within 1 hour towards zero, while being distributed in the animal tissues (volume of distribution, Vd=3722 ml/Kg) (Supplementary Figure 2). In previous tests, 50–55 mg/Kg PJ34 applied I. P. or I. V. similarly eradicated cancer cells. Treatments with 15 and 30 mg/Kg PJ34, administered either I. V. or I. P. were less efficient. A course of three weeks treatment with PJ34 including 8 or 14 PJ34 applications, provided the best results. Shorter I. P. or I. V. treatments with PJ34 were less efficient.

At the end of the study, all mice were euthanized by CO_2_ inhalation. Tumors were excised. Their weight and volume were measured, and they were prepared for immunohistochemistry.

### Histology and immunohistochemistry

Excised tumors were fixed in 4% paraformaldehyde and embedded in paraffin. 5 μm sections of the whole tumor were prepared. Several sections (3–6) were incubated for 1 h with 3 primary antibodies specifically directed against one of three arbitrarily depicted human proteins: human kinesin HSET/KifC1 (Mouse Monoclonal, sc-100947, Santa Cruz), human Ku-80 (Rabbit Monoclonal, 2180T, Cell Signaling), and anti-human leucocytes antigen HLA-A (Rabbit Monoclonal, Ab52922, Abcam). In addition, sections were immunostained for Smooth Muscle Actin (αSMA) with mouse monoclonal antibody (202M-94, Cell Marque) that labeled normal fibroblasts infiltrated in the tumors. Each protein was labeled in 3-6 different sections of each tumor. Immunolabeling was performed at room temperature in a humidity chamber. Immunolabeled tissues were incubated with secondary antibody (HiDef Detection Polymer, 954D, Cell Marque, Rocklin, CA, USA). A peroxidase substrate kit (SK-4100, Vector Labs, USA) was used as a chromogen (brown), and hematoxylin was used as a counterstain. In immunofluorescent labeling, two secondary antibodies were used: Alexa Fluor 488 Donkey anti Mouse (715-545-151, Jackson Immunoresearch) –green, and Cy3™ Donkey anti Rabbit (711-165-152, Jackson Immunoresearch)-red. A 6-week-old murine testis was used for positive control labeling of antibodies directed against the human proteins. The exposure to secondary antibody without labeling with first antibody was used as a negative control labeling. Usually 8 fields in each slide were analyzed. Immunolabeling quantification and statistical analysis were performed by ImageJ, as described before [31].

### Measurement of necrosis in PANC1 tumors

Slices in each tumor were stained by Haemotoxylin and Eosin dyes. Haemotoxylin is a basic dye that stains nuclei in purple. Eosin stains cellular cytoplasm in pink. Dead cells were detected in the slices by light microscope at high resolution. Necrosis was quantified as percentage of the total cell count in arbitrarily selected fields in each slice.

## SUPPLEMENTARY MATERIALS


