# Correction: The phenanthrene derivative PJ34 exclusively eradicates human pancreatic cancer cells in xenografts

**DOI:** 10.18632/oncotarget.27440

**Published:** 2020-02-04

**Authors:** Leonid Visochek, Dikla Atias, Itay Spektor, Asher Castiel, Talia Golan, Malka Cohen-Armon

**Affiliations:** ^1^ Sackler Faculty of Medicine, Tel-Aviv University, Tel-Aviv 69978, Israel; ^2^ Sagol School of Neuroscience, Tel-Aviv University, Tel-Aviv 69978, Israel; ^3^ Oncology Institute, Sheba Medical Center, Ramat Gan 53621, Israel

**This article has been corrected:** Due to an error during image processing, frame 6 of tumor #1 (one of the control tumors) in Figure 4D is an accidental duplicate of frame 5. At Oncotarget’s request, the authors provided the original data. An original, correct Figure 4D is presented here. The authors declare that these corrections do not change the results or conclusions of this paper.

Original article: Oncotarget. 2019; 10:6269–6282. 6269-6282. https://doi.org/10.18632/oncotarget.27268

**Figure 4:**
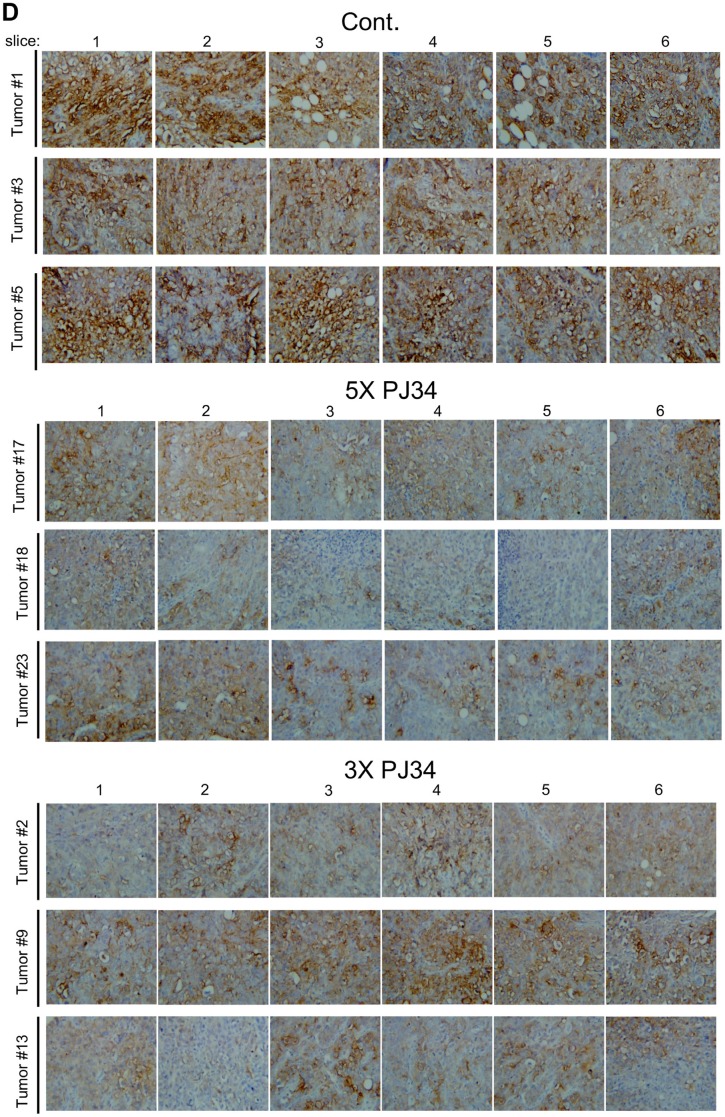
A massive reduction of human proteins in PANC1 xenografts in response to treatment with PJ34.

Three arbitrarily selected human proteins were specifically immunolabeled in PANC1 tumors developed in nude mice of 3 tested groups, control and treated with PJ34, 5 times, or 3 times a week (PJ34, 60 mg/Kg in 100 μl saline). Proteins were specifically immuno-labeled in the excised tumors, 30 days after the 3 weeks treatment with PJ34 has been terminated. Immuno-labeling with specific antibodies directed against human HSET/kifC1 (**A**), the c-terminal of human Ku-80 protein (**B** and **C**) and Human Leucocytes Antigen (HLA) (**D**) is displayed. In (**C**) fluorescent immuno-labeling of human Ku-80 (red) and the Smooth Muscle Actin (aSMA) (green; expressed in rodents and human fibroblasts) are displayed. (**E**) A quantitative analysis of the immuno-labeling indicates a reduction of 80–90% with a high statistical significance in the indicated human proteins (**A**–**D**) in tumors of PJ34 treated mice versus control mice. Immuno-labeling of aSMA was hardly affected by the treatment with PJ34. Control: red columns, PJ34 treated: 5 times a week-black columns, 3 times a week-grey columns.

