# Supplementary material for: The phenanthrene derivative PJ34 exclusively eradicates human pancreatic cancer cells in xenografts
Source: Oncotarget. 2019 Oct 22;10(58):6269–82. doi: 10.18632/oncotarget.27268 (PMC6817443; doi:10.18632/oncotarget.27268)
Supplement: Supplementary file 1 [file oncotarget-10-6269-s001.pdf]

The phenanthrene derivative PJ34 exclusively eradicates human pancreatic cancer cells in xenografts

SUPPLEMENTARY MATERIALS

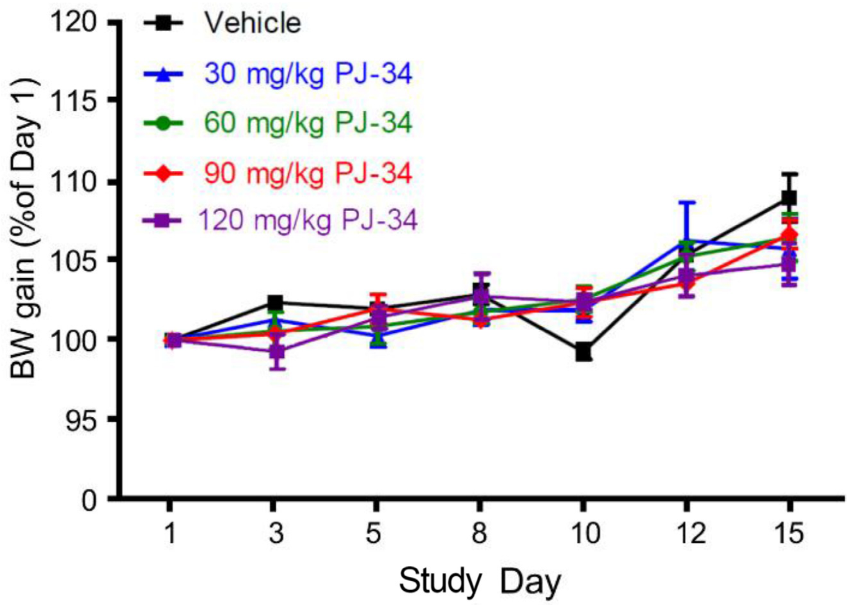

Supplementary Figure 1: Measured changes in the body weight (% of weight at day 1) of BALB/C mice treated with PJ34 (I.V.) at the indicated doses. Total 15 mice, 3 mice per group. Upper dose was limited by the limited solubility of PJ34 in water, 22 mg/ml).

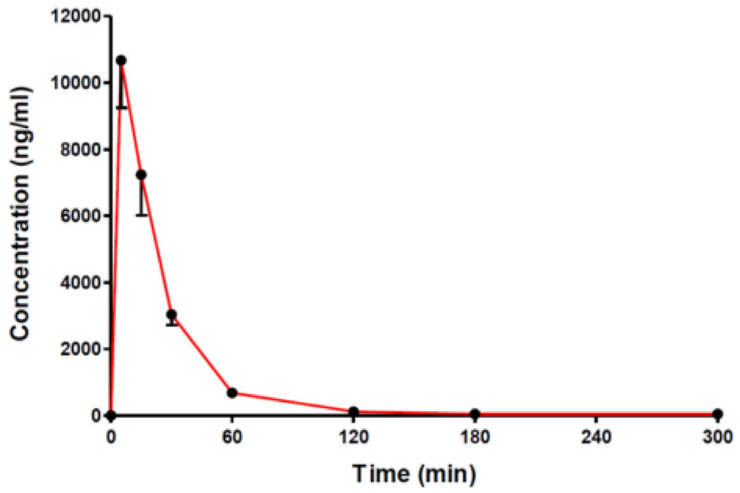

Supplementary Figure 2: Measured PJ34 concentration in the blood plasma of nude mice after I.V. administration.
